# Ethical wisdom in family planning programming

**DOI:** 10.3389/fgwh.2026.1779064

**Published:** 2026-08-21

**Authors:** Beniamino Cislaghi, Elizabeth Larson, Eloisa Montt-Maray, Marieme Fall, Lamiah Adamjee, Thais Gonzalez-Capella, Nour Horanieh

**Affiliations:** 1 Health Cluster, Institute for Development Studies, Brighton, United Kingdom; 2 School of Social Sciences, Makerere University, Kampala, Uganda; 3 The Wild School of Liberation Activism; 4 Center on Gender Equity and Health, University of California San Diego, La Jolla, CA, United States; 5 Department of Global Health and Development, Faculty of Public Health and Policy, London School of Hygiene and Tropical Medicine, London, United Kingdom; 6 Department of Epidemiology, Biostatistics and Occupational Health, McGill University, Montreal, QC, Canada; 7 Department of Family and Community Medicine, College of Medicine, King Saud University, Riyadh, Saudi Arabia

**Keywords:** family planning, ethics, reproductive justice, decolonisation, reproductive rights, SRHR, ethical framework

## Abstract

Despite the contemporary intellectual consensus within the family planning (FP) community on the need to implement rights-based interventions, little attention has gone into understanding when, why, and which rights-based interventions are ethically sound. Although we are in a time of increased attention to decolonisation processes on global health, current political climate is shifting the discourse on the need for decolonisation, reproductive justice and rights-based approaches in sexual and reproductive health. Hence, we offer this paper to begin the conversation on what questions can help the design of ethical right-based family planning grants and interventions. We suggest six interconnected principles governing these conversations, which we present separately to look at their specific validity: (1) compassion; (2) vulnerability; (3) voice; (4) authenticity; (5) agency; and (6) equity. Using these principles as a guide, we encourage actors across the family planning community to come together to hold courageous critical conversations on whether the resources invested in family planning interventions go beyond effectiveness to have ethics as the ultimate goal. We hope that the family planning community will build on and expand upon this framework, incorporating it into standards of practice as part of a broader transition toward self-reflection and transparency around intentions and actions.

## 1 Introduction

Is it worth our time considering if existing family planning programmatic efforts are ethical? In 1972, Callahan suggested in *Science* that the question on whether family planning interventions can both tackle excessive population growth and respect people's freedom is the wrong question, as there is no absolute answer for it (1). The real question, he suggested, should be: “what do they [human beings], in the end, finally value?”. In today's world, especially in the highly individualistic Global North-West, the public moral compass keeps in very high value respecting people's autonomy and choices, including when it comes to their sexual and reproductive health and desires. Most people socialising in this cultural intellectual milieu would keep women's reproductive desires in higher consideration than reducing global fertility rates. Yet, as activists and academics in the field recall, the discourse on women's desires in family planning had to evolve substantially to get to where it is today and to reach its current definition. Debates on this topic are not limited to modern times but have been a topic of discussion in ancient civilisations that had legal, religious and cultural frameworks on the right to choose when and how often to reproduce: autonomy is not a modern Western concept, although is highly valued and prioritised. However, the modern definition of reproductive autonomy had to go through several global debates over a number of decades. In 1974, the Bucharest World Plan of Action affirmed the right of couples and individuals to freely and responsibly choose the number and spacing of their children. In 1984, IPC in Mexico city affirmed the need to improve the status of women and influence family life and size in a positive way. However, it was not until the 1994 Cairo's International Conference on Population and Development, that a solidified global intellectual consensus was formed and family planning programmes were described as needing to prioritise respecting people's reproductive intentions and rights over achieving global fertility rates. The conference suggested that programmes that used exclusively targets of increasing modern contraceptive use risked promoting coercive interventions and clinical practices (particularly/disproportionally in the Global South) (2). Cairo's participants advocated instead for rights-based approaches that respected and promoted people's autonomy and desires, envisioning a world in which: “All couples and individuals have the basic right to decide freely and responsibly the number and spacing of their children and to have the information, education and means to do so” (UN, 1995). The conference was a pivotal point in moving ethics debates beyond whether abortion and infertility treatment should be part of family planning interventions, towards deciding the legitimacy of increasing contraceptive use. The 2012 Family Planning Summit in London, then, aimed to operationalise (at least in theory) the rights-based agenda through Family Planning 2020, a “global partnership to empower women and girls by investing in rights-based family planning” (FP2020), continued today in the efforts of FP2030.

Despite the contemporary intellectual consensus on the need to implement rights-based interventions, little attention has gone into understanding when, why, and which rights-based interventions are ethically sound. If anything, today the conversation on the moral justifications of family planning has given way to the discussion on the most effective operationalisation strategies. Yet, ethical shadows in family planning are all but dissipated by the apparent consensus on rights-based approaches. Not only is rights-based language often used in fundraising and not translated into practice, but there is also lack of consensus among family planning theorist on how that translation should actually happen (3). Together with others, we question that calling an intervention women-centred might be a guarantee of its ethical practice, especially when indicators of so-called right-based interventions are limited to uptake of modern contraceptive methods (4).

At this generative time of increased attention to decolonisation processes on global health (5), the conversation can be no further delayed. In this paper, we intend to generate heuristic motivation, opportunity, and language to discuss why, how and for whom family planning programmes are designed and implemented. This paper is intentionally conceptual and reflective rather than empirical, and we do not view the principles as universal truths. Instead, they aim to inspire discussion through reflections that act as a heuristic model for opening dialogue in an area where ethical frameworks are still under development. As such, we offer some clarifying distinctions for those non familiar with ethics and moral philosophy. Then, we suggest six ethical themes to guide the conversation on the design of ethical family planning grants and interventions. Finally, we offer some key concluding remarks on how the framework can be used and improved in the years to come.

## 2 Conceptual background

### 2.1 Ethical actions: process, outcomes and values

Moral philosophy is an extremely vast discipline, with many (and at times contradicting) theories of what makes an action ethical. For this paper, and knowingly engaging in an approximation, suffice to say that three broad streams of ethical thought exist: (1) procedural ethics; (2) outcome-oriented ethics; and (3) virtue ethics (6–8). Proponents of procedural ethics (e.g., Rawls) argue that ethical action is ensured when one follows specific rational processes to make decisions that hold moral relevance. Kant's categorical imperatives are an example of procedural ethics. His first imperative demands that every time one is to make a moral decision, they ask themselves whether it would be advisable that everyone did the same (9). Outcome-oriented ethics, in contrast, considers the (anticipated) consequences of one's action to evaluate whether that action is ethical or not. Outcome-oriented ethical approaches (e.g., Mill's utilitarianism: ensuring the largest good for the largest number of people) thus judges the ethical value of an intervention from the changes that an intervention is trying to achieve (10). An outcome-oriented ethical approach could, for instance, justify interventions that aim at reducing women's fertility on the basis that they will allow our species to survive in a planetarian environment with finite resources. Finally, virtue ethics (e.g., Confucius, Aristotle, McIntyre or Nussbaum) suggests that ethical action naturally follows out of the agent's virtuous qualities (e.g., equanimity or compassion) (11). For example, when a programme is designed by compassionate people, any programmes they design shall be ethical, as long as those people fully embody honest compassionate intentions in their work.

In addition to the tripartition above, another major conceptual distinction exists in the ethics literature: that between scholars arguing for the existence of universal moral values and those arguing against it. The distinction is key for the purpose of our paper and, more generally, for global health interventions: if universal and absolute moral values exist, cross-cultural ethical action will require embodying those values. Unfortunately, solid arguments exist against moral universalism (12). It would be futile to review those arguments here, as the debate does not lend itself to a definitive solution. Suffice to say that we adopt the stance of those who believe in the impossibility of universal moral judgement. That is, we believe in an ethics of care in which the responsibility of evaluating the morality of an action sits with the individuals performing the action itself.

In this paper, we chart a self-reflective process to invite individuals and institutions in the family planning community to engage in that process and take on their moral agentic responsibility. More specifically, we offer six principles pointing to critical ethical questions in family planning work. Our purpose is to invite grant making and implementing institutions to engage and answer those questions as they plan their work. We do not mean to be prescriptive with this framework, we don't hold the right answers; in fact, we believe these answers will vary in space and time. We instead intend to begin the conversation on how we can make sure that global investments in family planning are not only effective in achieving their goals, but also ethical in why and how they do so, and in the very goals they set for themselves.

## 3 Six principles for self-reflective ethical planning

In this section, we discuss six principles that we identified as potentially serving more ethical family planning programming (See Table 1). The first two (compassion and vulnerability) are virtues related to implementers' intentions. The second two (voice and authenticity) are processes, related to the programmatic procedures: how a programme is designed and implemented. Finally, the last two (agency and equity) relate to the outcomes that a programme might result in.

**Table 1:** Overview of six principles and their relevance, key question, and timing of operationalisation.

Ethical relevance	Principle	Key question	Operationalisation
*Virtues*	Compassion	What is the intention of the implementer in designing and implementing the intervention?	Implementer intention
	Vulnerability	How are implementers open and ready to learn from participants, without overriding participants’ values and self-help capacity?	Implementer intention
*Procedures*	Voice	Who is involved in making decisions about family planning interventions?	Programme design
	Authenticity	How transparently is information about the intervention shared with study populations and other researchers?	Programme design
*Outcomes*	Agency	Who has agency to make decisions about whether an individual uses or doesn't use modern family planning methods?	Programme outcomes
	Equity	How are resources distributed in the participating population?	Programme outcomes

### 3.1 Compassion

Despite a large and multi-faceted body of empirical and theoretical literature, most scholars in the field agree that compassion is an important source of moral judgement, one that should be always part of the processes through which ethical decisions are made (13–16). Several theories define compassion as a subjective feeling connected to the desire to help another person, one that emerges when paying attention to this other person's suffering. We feel compassion when we value the other as a peer of equal dignity and worth; that is, without the appraisal of the other's inferiority that, instead, is present when feeling *pity*.

The principle of compassion invites family planning actors to reflect on the ultimate intent of their interventions. We suggest four steps that grant makers, NGO workers and clinicians can follow to integrate compassion in their family planning work (See Table 2). The initial step relates to checking for possible compassion biases. The first is *identity* bias: human beings are much more likely to feel compassion towards people to whom they feel close or with whom they identify. The second is *numeracy* bias: not only do we feel more compassionate towards those who are similar to us, but we also naturally struggle to feel compassionate towards large numbers of people. And finally, *intergroup* bias: social categorisations (such as *us-and-them* narratives) create distance between people, limiting their compassion potential for each other (17). To counteract these biases, the literature suggests to, first, be aware of them, and then to both focus on previous adversities we experienced to feel the pain of others and engage in loving, kindness meditation exercises that reduce psychological stress, facilitating the recognition of the other person's pain (18–20).

**Table 2:** Institutional steps to integrate compassion in the design and implementation of family planning grants and interventions.

Step	Action point	Guiding questions	Possible operationalisations
Checking compassion biases	Acting upon identity bias	Who are those most similar to us in this intervention that we might more easily feel compassion towards?	Institutional self-reflection exercises
	Acting upon numeracy bias	To what extent are we being mindful of the lived experience and human existence of every single participant in the intervention?	Reminding staff members’ past adversities
	Acting upon intergroup bias	What are the existing narratives of suffering that might activate our compassion to certain specific populations?	Institutional routines including self and mutual caring opportunities
Setting compassionate intention	Generating compassion energy	How are we present to the lived experience of intervention participants?	Engage in person with (people who share characteristics with) intervention participants.
Actualising and evaluating compassionate intention	Holding compassionate processes	How are we ensuring desire to achieve outcomes does not sacrifice compassionate processes?	Implementing institutional policies that reward implementing compassionate processes
	Preventing compassion fatigue	How are we caring for overwhelmed staff members that might dissociate from suffering seeking refuge into cynicism?	Creating space for vulnerable engagement of staff members to carry out self-care check-ins.

The second step relates to setting compassionate energy and intent. Compassion energy emerges from the realisation of the interconnectedness of human beings. To generate compassion energy, we need to experience and being fully present to the *other*. In practical terms, this could mean visiting the place where the intervention will be carried out, listening to future participants' lived experiences and stories. Without an embodied relatedness to the other, it's nearly impossible to set compassionate intent. Without the presence of an embodied other, we distance ourselves from them, using technology as a way of disembodying us from the overwhelming compassionate feelings that might emerge when witnessing lived suffering, thus transforming meaningful compassionate work into “empty busywork” (21, 22).

The final step relates to actualising compassionate intent and evaluating its actualisation. To do so, organisations should strive to prevent compassion fatigue, the feeling of hopelessness and disengagement that can emerge when inner management of self or others' suffering is left unattended (23, 24). Self-caring check-in opportunities should be granted to those implementing compassionate interventions. Evaluating compassionate intent is also challenging, especially when it might generate unintended (but often unavoidable) harmful consequences. Because another's suffering produces a sympathetic response, as humans we tend to naturally repel responsibility of harmful outcomes, that is the possibility that harm might be consequences of the self's actions. Similar considerations result in sadness, distress or even fear (25). Evaluating compassionate intent, thus, becomes in itself a compassionate action, where people working in funding and implementing institutions are invited to take responsibility for the consequences of their choices in a safe constructive environment. Evaluating compassionate intent requires being honest with oneself and others about possible harm caused, a predisposition deeply connected to the principle of vulnerability, as we discuss in the next section.

### 3.2 Vulnerability

In the medical sciences, the word vulnerability is mostly used to refer to people's susceptibility to harm, an imperfection of nature that health researchers and clinicians are called to solve (26). Bioethicists further suggested that recognition of human vulnerability (either biological, structural, or relational) triggers a moral duty to protect the vulnerable, with several moral theorists suggesting that witnessing vulnerability is one of the very motivators of moral action. Recent research in social work instead challenged the idea of vulnerability as a human flaw (15, 27–29). Brown, for instance, contended that “The perception that vulnerability is weakness is the most widely accepted myth about vulnerability and the most dangerous” (30, p. 64). In this paper, we refer to vulnerability as the capacity to connect with one's flaws, fears, and suffering, and make them public so that they can become a source of power and resilience.

The principle of vulnerability thus invites family planning actors to engage in a revolution of humbleness, so to speak. From a colonial paradigm in which trained experts from the world's intellectual elite know what is best for people, to one in which those in power only become facilitators of discovery of ultimate knowledge about what people want (31). If the concept of vulnerability were to be summarised in a sentence, it would probably be a very Socratic admission such as: We do not know. In a constantly shrinking funding pool for international aid (32, 33), international NGOs experience unprecedented competition for their survival that in turn push unrealistic commitments to achieve family planning outcomes with limited resources (34). International funding agencies would naturally respond to greater and cheaper proposals with an increased demand. The funding tragedy that naturally followed the financialisation of aid is now fuelling resource competition among implementing organisations and is dramatically shrinking the space for collective rethinking of failed approaches (35). As Roy put it: “[The] Funding [industry] has fragmented solidarity in ways that repression never could” (36, p. 645). We live in a world where showing vulnerability is often difficult or financially dangerous, yet we—the authors of this paper—see no way forward than welcoming that vulnerability for global growth and advancement.

Institutionally, we suggest that welcoming vulnerability might be embodied in two steps (Table 3). First, we suggest that institutions create internal systems and incentives to reduce pressure to achieve predetermined outcomes. They could, for instance, integrate process-oriented worldviews in their grants and interventions, and reward officers and future grantees who invest time into understanding how to do so. Rather than striving to achieve predetermined outcomes as a measure of success, ethical family planning programmes could focus on the value-oriented processes that they facilitate among participants, and how these processes nest into and intersect with long-term change waves. Participants could then report on outcomes that mattered to them (37). Second, we suggest that partners in the international family planning community could reveal their uncertainties and failures to each other (both among peers and with grant makers), building an open-source community of people ready to fail, to make mistakes, to admit that, for instance, Western medicalised programmatic approaches can, at times, be inadequate and inappropriate. Western understandings of gender, for instance, have been recently shown to be highly inadequate to make sense of power relations among indigenous populations, and—if used blindly as a way of seducing Western grant-makers into distributing funding—might be harmful (38–41). Being vulnerable in global health work requires preparing to question the deepest assumptions motivating that very work, such as for instance, that all humans attach the same value to life expectancy, to freedom, to autonomy or choice. It requires learning from participants and trusting that their desires might be conceptually very far from those of the grant makers and implements. As an example, several scholars have criticised the assumption that all women would reasonably want to limit their fertility, and yet family planning programmes are designed with this very assumption in mind (42). Being vulnerable thus requires rejecting the “obscene caricature” (43) of Western models as valid globally and engage in cross-fertilising conversations that challenge basic mental models and assumptions affecting design of grants and interventions. Yet, as we mention in the next section, being open and reflective about one's (both personal and institutional, practical and theoretical) limits and ignorance is but the first part of an ethical partnership for the collective good.

**Table 3:** Institutional steps to integrate vulnerability in the design and implementation of family planning grants and interventions.

Step	Action point	Guiding questions	Operationalisation
Rethinking institutional priority of outcomes	Prioritize value-oriented processes over outcomes	How are we balancing institutionally the importance of democratic participatory processes with achievement of outcomes?	Evaluating and reassessing internal and external system of incentives
	Creating institutional culture open to failure	How do we become more open to discussing failure across institutions to learn from each other and improve?	Discussion and sharing of experiences across implementing institutions and funders
	Sharing interventions evaluation with local populations in participatory and mature ways.	How can our institution communicate to participants the results of our intervention, asking their opinion and feedback?	Including participant-led evaluations and validation of external evaluations as part of grants and interventions
Learn from intervention populations	Integrate medical knowledge with thick anthropological understanding of the existing local cultural worldviews and relations	How do our institutional biases (especially in Western-based institutions) affect our predisposition to certain approaches over others	Implement participatory methods during intervention development

### 3.3 Voice

International aid organisations, including United Nations agencies, global philanthropic foundations, and international Nongovernmental Organisations are, for the most part, largely undemocratic institutions (44). Their leaderships hold influence, resources, and power, without the political accountability that comes from having been elected. The principle of voice invites these institutions to engage in accountable, democratic processes of mutual understanding for global collective improvement.

Increasing people's voice and representation in strategic decisions with programmatic resonance requires increasing their participation in the organisation's functioning. Perfunctory and tokenistic participatory methods have been heavily criticised for being despotic (45); true processes for people-led development, instead, have shown effective capacity to invert unhelpful colonial power hierarchies and increase citizens' voice in the critical decisions that affect their live (46–49). We suggest that grant-makers and intervention designers could engage in a version of the following reflection and action steps (See Table 4 below). The first of these steps is to reflect on the extent to which they intend to co-create grants with local citizens. Working with citizens can help prioritise services based on their real (as opposed to assumed) needs, but it must be approached with openness to break siloes. While services might be organised in siloes, citizens' lives require bridging across those siloes to meet them in the complexities of their daily lives (for instance, offering interventions that both help them achieve their SRH needs and escape situations of violence). Following the collective design of those interventions, international organisations should discuss the extent to which they intend to co-implement and co-evaluate them. Genuine collective implementation and collection of knowledge also increases the likelihood that the learnings have immediate practical application that is relevant to improving the intervention (50–53).

**Table 4:** Institutional steps to integrate voice reflections in the design and implementation of family planning grants and interventions.

Step	Action point	Guiding questions	Possible operationalisations
Collaborative grant making	Review existing organisational policies hindering or facilitating co-creative grant-making	Who traditionally decides grant goals, strategies, and priorities within our organisation?	Conduct internal review of organisational policies
		How are internal institutional grant-making processes allowing pre-grant co-creational research that help set people-led priorities?	
	Conduct explorative participatory action research with population of future grants	How do participants in future interventions in the region of interest prioritising across their needs?	Conduct participatory formative research at grant-making state
		What are populations preferences when it comes to their fertility and sexual health?	
Collective implementation	Facilitate partnerships between implementers and populations	Which cultural, social, and economic resources exist within the local population that can be supported during the delivery?	Incentivise the creation of intervention management units within the participating population
Collaborative evaluation	Check incentives that might be biasing data collection or interpretation	What hopes and wishes for interventions outcomes are being unconsciously put on implementers and participants?	Conduct periodical research with participants and implementers on the perceived desirability biases they experience.
	Incentivise collectively led monitoring and evaluation processes	How do participants evaluate the intervention with regard to its holistic contribution to a happier life?	Integrate non-medical participant-centric measures to evaluate intervention processes holistically

### 3.4 Authenticity

Despite its several definitions, with the term authenticity most of the literature refers to the coherence between one's internal values and their external expression. In the understanding that we put forward in this paper, we suggest that the principle of authenticity invites family planning actors to be (1) true to what motivates their action (so not to sacrifice their values); (2) transparent with participants about those actions; and (3) honest with peers about the processes and results related to their interventions. We suggest three steps to embody authentic processes in family planning work (Table 5). The first involves creating organisational processes and strategies based on values, rather than outcomes. This could be done, for instance, by doing value-based recruiting, onboarding, and performance reviews. By inviting staff members to be truthful to the organisational values, rather than exclusively achieving predetermined outcomes, international family planning organisations have an opportunity to create an ethical organisational culture that integrates an intention to be aligned with moral, rather than exclusively pragmatic, ideal end states for the world. Secondly, family planning interventions should be transparent with participants from the start about the goals of those interventions and suggest alternative sources of information for those interested in opinions that dissent with those of the implementers. Finally, we suggest that institutions create incentives to communicate transparently (both within and outside of the organisations) their values, processes, and results. Recently there has been a call in global health to share evaluation data publicly. We encourage details involved in data collection, intervention design, data analysis and interpretation should also be reported, including the ones that could bring the whole process into question. Sharing failure is a tremendous and underutilised opportunity for collective growth and improvement (54). An open-access approach to problem-solving in family planning interventions, where participants, colleagues, researchers are invited to discuss and co-operate in imagining pathways around the experience of failure, would be greatly beneficial, despite the threat that they comprehensibly pose to the personal and institutional egos of the implementers. The purpose of international convenings should not only be to share great intervention results but also to create opportunities for practice problem solving and ethical reflections on what to do when that happens.

**Table 5:** Institutional steps to integrate authenticity in the design and implementation of family planning grants and interventions.

Step	Action point	Guiding questions	Possible operationalisations
Organisational value-action synchronicity	Create value-informed recruitment processes	How are our organisational values aligned with the individual values of the people we recruit?	Institutionalise the importance of both values and skills in recruitment
	Create value-informed strategies and reviews	How are our grants and interventions aligned with organisational values?	Integrate value-based questions within evaluation of institutions and reviews of organisational policies
Transparency with participants	Ensuring clear communication of implementers’ desired outcomes	To what extent are participants aware of the success measures of this intervention?	Integrate human conversations at the beginning of any intervention with participants about the desired outcomes and implementers’ incentives
	Ensuring participants know where to find information that diverges from the one received by the implementers	To what extent are participants alerted to the possible biases in our work?	Collaborate with organisations that hold diverging views to the ones of the implementers for the collective caring of participating populations
Transparency with other family planning actors	Communicating outcomes and shortcomings transparently with family planning community	How transparent and honest are we being in sharing our data and interpretation biases	Document and process successes and failures at all stages of the grant and intervention design, implementation and evaluation.

### 3.5 Agency

The concept of agency has been widely discussed in the social and health sciences. At its simplest, the term refers to people's capacity to decide to act and act (55), or to decide not to act and do so (56). As critical health researchers have observed, agency can be both individual (one person's capacity to act freely) or collective (the capacity of a group, an organisation, or a system to act in ways that help them achieve their goals). Family planning actors could integrate considerations on agency in their work could do so by following four steps (See Table 6).

**Table 6:** Institutional steps to integrate agency reflections in the design and implementation of family planning grants and interventions.

Step	Action point	Guiding questions	Possible operationalisations
Setting agency intention	Decide whether the intervention should promote agency or uptake	Is this investment/institution hoping to help people attain what they desire, or to achieve specific reproductive targets?	Pre-design institution reflections
Agency decisions (related to population)	Selecting priorities for agency expansion	Whose agency this institution/investment/intervention intends to promote and why?	Pre-design institution reflections
	Reflect on whose choices the intervention might limit	Whose agency this institution/investment/intervention might limit and why?	Pre-design institution reflections
Agency decisions (related to method)	Decide which methods should the intervention promote	Is there a method of preference that this institution/investment/intervention will be promoting and why?	Pre-design institution reflections
		What resources exist in this institution/investment/intervention to revert choices of contraception?	Pre-design institution reflections
Creating and using mixed-methods indicators of agency	Set relevant success indicators	What indicators of choice will this institution/investment/intervention use to guarantee that every single person reached by the intervention made an informed decision?	Pre-design institution reflections

Firstly, family planning actors should decide whether they intend to defend, promote and expand participants' agency at all. Despite the contemporary large consensus that family planning programming should defend women's rights, some actors prioritise global population reduction (57), thus choosing to ignore people's own choices. For reasons provided later, several scholars argued that using the unmet needs measure to make the two views (defending reproductive rights and reducing reproductive dividend) elide into one is a loophole rather than an effective solution to the conundrum (70). Many commentators also argued that guaranteeing women free and informed contraceptive choice is extremely challenging, especially when: (1) certain method are not available where they live (71); (2) service providers are biased towards people using modern methods (72, 73); and (3) service providers are under pressure to achieve targets for use of a specific method (74). These are not hypothetical speculations. With regard to providers' biases towards modern methods, scholars have remarked how Western favouritisms towards biomedical technologies tend to dismiss the possibility to adapt other “traditional” solutions that have been proven to work (58) (75, 76). And related to the third point (on providers' biases and pressures), researchers documented both that clinical removal of Long-acting reversable contraceptives (LARCS) and permanent methods is often underfunded and that providers can discourage (or even forbid) women from proceeding with their removal anyway (74).

Secondly, we invite family planning implementers to reflect on whose agencies they intend to promote and why. Traditionally, many family planning activists and scholars suggested that women should have full autonomy over the decisions that affect their bodies. Because material (e.g., limited services) and social barriers (e.g., gender norms) often limit these women's choices, interventions should aim to either remove or help women navigate those barriers (59).

The third step is to consider whose agency the intervention is limiting. In addition to the case of partners who are not allowed to partake in women's decisions, helping women make completely autonomous choices might reduce the role of other actors, e.g., their in-laws, parents, siblings, friends, or religious leaders. This idea of women being sole decision-makers has been met by some researchers as an imposition of Western individualist values in non-western societies, where communal decision-making might be more culturally appropriate (60, 61). Of course, the complexity of the arguments lies in the fact that these other people can also exert coercive pressure in ways that dim or erase women's voices (77).

Lastly, considerations on people's agency arise when deciding what success indicators said interventions should adopt. Targets focused only on reproductive outcomes might demean and misrepresent women's agency. Even if one supposes that all women in the world desire family planning, similar indicators would still be inadequate: women's reproductive wishes are not reducible to binaries such as having or not have a child (58). Women might be ambivalent, for instance, or their choices might change according to age, partner they are with, the death of their parents, or their economic situation, just to cite a few examples. New indicators are thus needed to capture women's and men's individual and collective agency in family planning (58, 62–64); we suggest family planning actors to invest in developing and adopting similar indicators.

### 3.6 Equity

An intersectional equity approach aims to ensure just distribution of family planning resources and services to meet the needs of people in different sectors of society. An equity analysis invites family planning actors to examine unfair unbalances in how people access family planning services. Examining inequity patterns across different socioeconomic dimensions in 64 countries (65), found that family planning services tend to be more accessible for (1) women of (2) higher economic status, (3) living in urban areas, (4) with at least secondary education, and (5) aged 35 or older. Other examples of relevant inequities include the fact that most family planning interventions and indicators used to measure success of programmes are designed for married or in-union women, ignoring, at many times, the needs for unmarried, people with disabilities, adolescents, young people as well as men (4, 66). Often the needs of people with disabilities are also ignored, due to biases about their contraceptive desires, needs and issues of accessibility in the family planning infrastructures (67, 68). Programmes may also be less concerned with providing access to services to those living in the most difficult-to-reach communities, focusing instead on more populated areas that are easier to access. None of these choices is inherently unethical. Take, for instance, the last example on how residence can affect availability of services. While controversial, the choice to focus on easy-to-access might be ethical from a utilitarian standpoint: resources would be better spent reaching a higher number of people than wasting resources on reaching the most vulnerable. Yet, justice activists might fiercely (and with good reason) disagree.

There exists a large body of literature on how to ensure an equitable distribution of family planning allocation, with some excellent examples produced in the last 40 years that literature can provide better practical steps of what we could summarise in a table for this principle. Yet, here, we provide some suggestions on the equity-related questions that family planning actors might want to reflect upon as they plan their work (see Table 7). Firstly, family planning actors could decide the criteria that they intend to follow in their work: who do they intend to serve, and why? They might, for instance, focus specifically on disabled or hard-to-reach populations. Secondly, they might want to look at historical patterns of resource distribution in their institution: who have been the main recipients of intervention resources, and why? The analysis might highlight that distribution of the institution's resources have been traditionally, but unintentionally, skewed towards a given population (e.g., married able-bodied women aged 15–49 and living in urban settings). Third, they can agree on periodically-revised resource allocation criteria to ensure equity in (1) how people (including those living in remote under-serviced areas of the world) can access SRHR services, and (2) how service allocation is prioritised across communities on different intersections of disadvantage.

**Table 7:** Institutional steps to integrate equity reflections in the design and implementation of family planning grants and interventions.

Step	Action point	Guiding questions	Possible Operationalisations
Understanding Population priority	Decide priority population	In case of conflicting priorities, who will the organisation decided to prioritise and why?	Pre-design institution reflections
Examining Funding and Intervention Patterns	Review internal funding patterns	Whose interested has the organisation been prioritising in its funding and intervention patterns? Was this a conscious strategic decision or a historical occurrence?	Grant-making or proposal writing agenda
	Review global funding patterns	Whose interested are currently being served the most in existing funding and intervention patterns? How is this organisation willing to align or diverge from those?	Grant-making or proposal writing agenda
Considering Effort Rotation	Decide possible rotation on populations and services to be supported	Is there a need to change who, where and when this organisations’ funding and interventions are serving foster greater equity?	Structure value-checking points to consider rotation of funding or implementation efforts
		Is there a need to rotate or change how the efforts of this organisation are prioritising service allocation across different communities?	Pre-design institution reflections

## 4 Conclusion

Women are dying, right now, due to the preventable consequences of unplanned and excessive pregnancies. Relatively simple medical solutions could prevent these deaths. The temptation to save these women's lives by convincing them to adopt medical family planning mthods is understandable. Yet, the desire to spare these women the harmful consequences of having many children has pushed many interventions to target specific reproductive outcomes, as opposed to developing methods and models to ensure that they help people (and their families) achieve lives that matter to them. This paper set out a framework to support family planning programmers to engage in a process of ethical reflection. The themes (or principles) we discussed in this theoretical framework are interconnected, interlocking in the practicality of life that no framework can fully capture in its changeable complexity. Still, we also acknowledge that tension exists within the principles. For example, “compassion” may conflict with “equity” in that prioritizing immediate suffering relief may not align with resource allocation to the most marginalized groups. Additionally, “agency” could clash with “voice,” making it difficult to promote individual choice while simultaneously honoring communal decision-making processes. It is important to consider these tensions when deciding how and when to incorporate the principles into interventions, since they serve as a guiding tool rather than a prescriptive approach. We hope that in the future the family planning community will build on and expand this framework, incorporating it into standard practice as part of a broader move towards of self-reflection and transparency about their intentions and actions. To encourage actors across the family planning community to come together to hold courageous critical conversation on whether the resources invested in family planning interventions go beyond effectiveness to have ethics as the ultimate goal. We hope that the family planning community will build on and expand upon this framework, incorporating it into standard practice as part of a broader transition towards greater self-reflection and transparency of intentions.

## Data Availability

The original contributions presented in the study are included in the article/Supplementary Material, further inquiries can be directed to the corresponding author.
